# A novel inpatient PA staffing model for a community hospital

**DOI:** 10.1097/01.JAA.0000795020.20041.2e

**Published:** 2021-12-23

**Authors:** Danielle Bendicksen, Chaya Mangel Pflugeisen, Franchot van Slot

**Affiliations:** **Danielle Bendicksen** practiced as a hospitalist PA at MultiCare Health System in Tacoma, Wash. **Chaya Mangel Pflugeisen** is a research scientist at the MultiCare Institute for Research and Innovation in Tacoma. **Franchot van Slot** is a hospitalist physician and medical director of the Inpatient Advanced Practice Provider Fellowship at MultiCare Health System and an adjunct professor in the PA program at Pacific University in Forest Grove, Ore. The authors have disclosed no potential conflicts of interest, financial or otherwise.

**Keywords:** physician assistant, PA, NP, hospitalist, staffing, model

## Abstract

**Objective:**

We sought to create a novel physician assistant (PA) and physician hospital medicine co-management strategy, employing a 3:1 PA:physician structure, under which the physician oversees all PA patients, but without a separate independent panel.

**Methods:**

This is a retrospective cohort pre-post design, comparing metrics for a traditional physician-only hospitalist model with a PA-physician team model. Outcomes included length of stay (LOS), readmissions, discharge destination, patient satisfaction, and in-hospital mortality.

**Results:**

LOS for patients under the PA-physician model (74 hours) was lower than for the physician-only model (83 hours; P < .001). The PA-physician model team discharged more patients home than to another facility (PA-physician 77.6%, physician-only 74.3%; P = .03). Thirty-day readmissions were about 10% (P = .97) and patients reported respectful treatment in about 80% (P = .53) of cases in each cohort.

**Conclusions:**

Our 3:1 PA-physician model team showed equal to superior outcomes compared with the physician-only hospitalist model.

Growing numbers of physician assistants (PAs) are working in hospital medicine. PAs and NPs were represented in more than 75% of US hospitalist groups as of 2018.^1^ PAs are considered a cost-effective means to enhance the reach of hospital patient care.^2^ Studies have shown that PAs can be successfully integrated into hospitalist services.^2^-^5^ However, the optimal working model for hospitalist services and the PA-physician relationship has not been determined in terms of outcomes, patient safety, and patient satisfaction.^6^

Various medical groups incorporate PAs in numerous and different practice models. Some groups function in a 1:1 PA:physician dyad in which the PA often sees a subset of the physician's lower-acuity patients and coordinates care with the physician.^4^ Some teaching hospitals employ PAs to complement resident physician teams; attending physicians oversee teams of residents and PAs.^5^,^7^ The optimal model and PA:physician ratio have not been determined. At our nonacademic community hospital, we instituted a PA-physician co-management strategy that is reminiscent of the resident team model.

Our PA-physician model employed teams comprising three PAs and one physician. Each PA in the model was primarily responsible for about 12 patients per day and collaborated with the physician. Patients were allocated to PA teams independent of acuity, and included critically ill patients in the ICU. The physician rounded on each patient and reviewed plans and PA chart notes. The physician in our model did not see a separate subset of patients independently, as may be similar to an attending physician at an academic medical center. Potential benefits of this PA-physician model include patients being seen by two skilled clinicians working as a team to care for them, while not compromising cost. Further, PAs in this model see fewer patients per day than a physician rounding independently, which may afford them more time to thoroughly review the medical case, focus on care coordination, and patient education. Meanwhile, the physicians in the PA-physician model are released from writing full notes and executing much of the care coordination. The physicians are therefore able to focus more on complex medical cases as the need dictates.

Similar PA and NP models have been compared with resident physician teams at teaching hospitals and in rural or short-stay units in terms of length of stay (LOS), mortality, readmissions, and patient satisfaction; however, this model has yet to be compared with a traditional hospitalist physician-only model.^3^,^8^ Therefore, we sought to evaluate our PA-physician model in comparison with the traditional physician-only hospitalist model with respect to standard metrics that hold value for medical groups and patients: LOS, mortality, 30-day readmission rate, next site of care, and patient satisfaction scores. We hypothesized that the PA-physician model would be noninferior to the physician-only model and could serve as a viable model for hospitalist practice in community hospitals.

## METHODS

Our healthcare system's institutional review board approved this study as exempt.

### Setting

The study was performed at a 136-bed nonacademic community hospital with a 6-bed ICU in Tacoma, Wash., a mid-sized city in the South Puget Sound region. With an effective capacity of about 60 inpatient beds, the study facility acts as a community sister hospital to a larger, 437-bed tertiary care center. Both hospitals are part of the South Puget Sound's largest healthcare system, which has five hospitals in the region as well as dozens of primary care, urgent care, and specialty clinics.

### Model

Before starting the PA hospitalist model, we initiated a 1-year PA inpatient medicine fellowship program in 2015 with the goal of introducing highly trained PAs into the hospitalist group. This practice is consistent with a growing trend toward postgraduate PA hospitalist training to supplement the variable but often limited PA inpatient training during standard graduate education.^2^,^9^ Before 2016, and before the introduction of rounding hospitalist PAs, hospitalist physicians were responsible for about 15 patients each day on 1-week (7-day) rotating shifts, Saturday through Friday. In 2016, our PA-physician model started staffing with the first graduated class of PA fellows; the team worked every other week, with the alternate weeks continuing with the physician-only model.

Average daily patient census for the PA-physician model was constructed to be salary-neutral compared with the traditional physician-only model. Although medical groups may seek to achieve financial savings by diversifying their hospitalist team with PAs, this was not our immediate objective. Our first objective was to determine clinical safety, viability, and quality of a new model. Therefore, we attempted to keep the overall patient census to hospitalist salary ratio neutral. This may be broadly applicable to other medical groups also seeking to diversify their teams and develop thumbnail working models. Salary neutrality was calculated by first adding up the total salary of the PA-physician team (in our case, three PAs and one physician). This team salary was divided by one physician salary, for a salary ratio of 2.4. We multiplied the salary ratio of 2.4 by the expected physician census of 15 patients/day to give an expected PA:physician team census of 36. This is the number of patients who would need to be seen if the same salary cost was used to pay for physicians alone. The expected PA-physician team patient census was then divided equally between three PAs and yielded an average of 12 patients per PA. We used starting salary calculations as the basis for this model, despite the fact that many of the physicians in the group had more than 10 years of experience. We did not factor in bonuses or benefits but doing so does not change the ratio and favors overall cost benefit. This ratio method proved useful in model design and in day-to-day balancing between solo physician panels and PA-physician team panels between the two sister hospitals managed by the medical group. In subsequent years, this ratio system also flexibly allowed for similar census balancing with two PAs per physician or the more traditional one PA per physician team dynamic, as need arose.

In 2017, the second PA fellowship cohort finished training and was employed to cover the hospitalist service of the alternate week in lieu of physicians only. With the exception of scheduled breaks for the two PA-physician teams, during which time physician hospitalists filled in, the hospital was continually staffed under the PA-physician model in 2017.

### Data collection and analysis

This is a retrospective cohort study comparing physician-only and PA-physician hospitalist models with respect to patient-important outcomes, such as LOS, mortality, readmission, discharge destination, and patient satisfaction scores. Using a pre-post design, we compared the two staffing models by analyzing 2017 PA-physician discharge data, 2015 physician-only discharge data, and patient demographics such as age and ethnicity. We also compared groups by primary admission diagnosis severity weights using nationally standardized All Patient Refined-Diagnosis Related Groups (APR-DRG) to assess the severity of various diagnoses, which ultimately are tied to reimbursement.^10^ Data were sorted based on the discharging clinician, and all physician-only model discharges in 2017 were excluded from analysis. Observation admissions were excluded because observation stays do not include relevant information such as APR-DRG severity or readmission data. We also were unable to capture data on consultations because the data were sorted by discharging clinician, which by definition would be from the primary service rather than consultants. Outcome data were extracted from QlikView applications that are built and maintained by the healthcare system. These applications aggregate data from patient encounters captured by the electronic medical record. LOS was calculated in hours. Mortality, which was not risk-adjusted, was defined as death during the hospitalization. Readmission was defined as return to inpatient status within 30 days of hospitalization.

Satisfaction data were tabulated from Press Ganey surveys sent to patients following hospital discharge. We used 2015 and 2017 Press Ganey results for the physicians in the PA-physician model. Press Ganey survey results are attributed to the billing clinician at discharge, and the physicians in the PA-physician model were the billing clinicians during the study period. Two physicians oversaw more than 80% of the PA-physician model discharges in 2017 and were therefore included in the analysis for 2017 satisfaction data. The few other physicians working in the PA-physician model were not included to avoid data contamination because they also cared for patients in 2017 under the physician-only model. The two physicians who most commonly worked with the PA-physician model in 2017 did not work under the physician-only model at this hospital. Five of the six PAs in the 2017 cohort were graduates of the system's PA fellowship. The one PA who did not come through the fellowship had several years of experience as a PA, as well as previous hospital medicine experience.

Univariate analyses were conducted to evaluate baseline characteristic differences between the discharges executed under the physician-only model in 2015 and those that were made under the PA-physician model in 2017. After performing visual inspections of boxplot distributions for continuous variables, measures of central tendency and dispersion were compared for the two groups; normally distributed variables were evaluated using means with standard deviations (SD) and compared using *t*-tests. Markedly skewed distributions were evaluated using the median with interquartile range (IQR) and the Mood median test. Categorical variables were evaluated using contingency tables and compared by hospitalist model (PA-physician and physician-only) using *chi*-squared tests. Univariate outcome analyses comparing the physician-only with PA-physician models were assessed using the same analytic approach. Variables that achieved significance (*P* < .05) in the univariate models were further assessed using multivariate linear regression for normally distributed continuous outcomes, quantile regression (set at the median) for markedly skewed continuous outcomes, or logistic regression for binary outcomes. Multivariate linear models were adjusted for clinically relevant and potentially confounding covariates chosen using forward/backward selection with Akaike information criterion. All data analysis and visualization were performed in the R statistical software environment (Vienna, Austria).

## RESULTS

The physician-only hospitalist model in 2015 had 2,296 discharges that met criteria for study inclusion, compared with 1,828 eligible discharges in 2017 under the PA-physician model, for a total of 4,124 discharges included in the analysis (Table 1). Mean patient age at discharge under the physician-only model was 66 ± 19.7 years, compared with 64.7 ± 19.3 years for the PA-physician model (*P* = .03). Median APR-DRG severity weight was 0.85 (IQR = 0.59) for those discharged under the physician-only model, compared with 0.95 (IQR = 0.74) for the PA-physician model discharges (*P* < .001). Both groups were about 58% female (57.8% physician-only model versus 58.7% PA-physician model; *P* = .58) and the self-reported ethnic composition of the two groups was similar (80.5% and 79% White, 10.5% and 11% Black; *P* = .45). These were evaluated to demonstrate consistent demographics and catchment area over the study design. However, the proportion of Medicare/Medicaid patients did differ significantly between the two models, with 68.6% of the physician-only model discharges insured by Medicare/Medicaid compared with 64% of the PA-physician model discharges (*P* = .002).

**TABLE 1.** Baseline characteristics between physician-only and physician-PA model discharges

	Physician-only model (2015)		PA-physician model (2017)		Total
Discharges (% of total hospitalist discharges between 2015 and 2017)	2,296 (55.7%)	1,828 (44.3%)	4,124
Age (years)	**Mean (SD)**	**Range**	**Mean (SD)**	**Range**	***P* value**
	66 (19.7)	18-102	64.7 (19.3)	18-103	.03
APR-DRG severity weight	**Median (IQR)**	**Range**	**Median (IQR)**	**Range**	***P* value**
	0.85 (0.59)	0-7.7	0.95 (0.74)	0-7	<.001
	**N**	**%**	**N**	**%**	***P* value**
Female	1,328	57.8%	1,073	58.7%	.58
Ethnicity					
Black	240	10.5%	202	11.1%	
White	1,849	80.5%	1,444	79%	.45
Other	207	9%	182	10%	
Medicare/Medicaid	1,574	68.6%	1,170	64%	.002

Median LOS for patients under the PA-physician model in 2017 (74 hours, IQR = 67) was 9 hours lower than that of the physician-only model in 2015 (83 hours, IQR = 69.3; *P* < .001). Given the severe skew of this distribution, we also evaluated the LOS distribution of those discharged within 7 days (LOS of 168 hours or less) of admission. The LOS effect remained significant after controlling for this subset of discharges, with the median shorter stay physician-only model LOS at 80.6 hours compared with 74.6 hours under the PA-physician model (*P* < .001). All-cause 30-day readmission rate was about 10% for each cohort (*P* = .97). Relatively few patients in either group died during their hospitalization, though this difference was statistically significant (2.3% physician-only model versus 1.3% PA-physician model, *P* = .02). The distribution of discharge location differed significantly between the groups, as well, with fewer physician-only model patients discharging to home, with or without home healthcare services (74.3% physician-only model versus 77.6% PA-physician model) and more to a skilled nursing facility (19.2% physician-only model versus 16.1% PA-physician model, *P* = .03; Table 2).

**TABLE 2.** Univariate outcome analyses

Outcome	Physician-only model		PA-physician model		*P* value
	Median (IQR)	Range	Median (IQR)	Range	
LOS hours; all discharges	83 (69.3)	4-2,486	74 (67)	8-1,520	<.001
	**Mean (SD)**	**Range**	**Mean (SD)**	**Range**	
LOS hours; LOS ≤ 168 hours	80.6 (36.2)	3-168	74.6 (36)	8-168	<.001
	**N**	**%**	**N**	**%**	
30-day readmissions (all cause)	242	10.5%	191	10.4%	.97
Died	53	2.3%	23	1.3%	.02
Discharge location					
Home/home health	1,665	74.3%	1,401	77.6%	
Skilled nursing facility	430	19.2%	290	16.1%	.03
Other	146	6.5%	114	6.3%	
Total	2,241	100%	1,805	100%	

All discharges were further evaluated using quantile regression and the subset of hospitalizations with LOS of 168 hours or less were further evaluated using linear regression, adjusting for relevant covariates. Median LOS was estimated to be 11.6 hours longer under the physician-only model for all discharges (95% CI 1.4-8.5, *P* < .001; Table 3A), and 7.5 hours longer under the physician-only model for discharges that took place within 168 hours of admission (95% CI 5.3-9.7 hours, *P* < .001; Table 3B). These models were corrected for APR-DRG severity weight, patient age, and discharge location; the model for LOS of 168 hours or less also included ethnicity. In analyses for all discharges and the shorter stays only, increasing APR-DRG severity weight remained associated with a significant increase in LOS (59.5 hours and 13.2 hours, respectively), as did discharge to a skilled nursing facility (42 hours and 26.1 hours, respectively). These results are based on the 3:1 PA-physician model managing a patient census such that the overall salary cost is consistent with the physician-only model.

**TABLE 3.** Linear models for LOS and discharge to home

(A) LOS hours—all discharges	Estimate	95% CI	*P* value
Physician-only model	11.6	1.4, 8.5	<.001
Age	0	0, -0.8	.42
APR-DRG severity weight	32.1	2, 15.9	<.001
Discharged to skilled nursing facility∗	34.1	2.4, 14.3	<.001
Discharged to other location∗	1.3	4, 0.3	.75
Discharged to morgue (died)∗	-19.6	13.4, -1.5	0.14
**(B) LOS hours—LOS ≤ 168 hours**	**Estimate**	**95% CI**	***P* value**
Physician-only model	7.5	5.3, 9.7	<.001
Age	0.1	0, 0.1	.02
APR-DRG severity weight	13.2	11.4, 14.9	<.001
White∗∗	-5.5	-9, -1.9	.003
Other ethnicity∗∗	-5.0	-9.9, -0.2	.04
Discharged to skilled nursing facility∗	26.1	22.8, 29.4	<.001
Discharged to other location∗	-6.4	-11, -1.8	.006
Discharged to morgue (died)∗	-21.8	-31.1, -12.5	<.001
**(C) Discharged home**	**aOR**	**95% CI**	***P* value**
Physician-only model	0.8	0.7, 0.9	.005
Age	0.96	0.96, 0.97	<.001
APR-DRG severity weight	0.62	0.6, 0.7	<.001
White∗∗	0.94	0.7, 1.2	.64
Other ethnicity∗∗	1.3	0.9, 1.9	.23
Medicare	0.78	0.6, 1	.05
**(D) In-hospital mortality**	**aOR**	**95% CI**	***P* value**
Physician-only model	1.89	1.14, 3.23	.02
Male	1.78	1.1, 2.9	.02
Age	1.07	1.05, 1.09	<.001
APR-DRG severity weight	2.05	1.78, 2.36	<.001
White∗∗	0.4	0.19, 0.91	.02
Other ethnicity∗∗	0.61	0.2, 1.78	.37

∗ Reference group: Discharged to home/home health

∗∗ Reference group: Black

Discharge to home with or without home health services was evaluated using a logistic regression model and adjusting for age, APR-DRG severity weight, ethnicity, and Medicare/Medicaid status. In this model, odds of discharge to home were significantly lower for the physician discharges (adjusted odds ratio [aOR] = 0.8, 95% CI 0.7, 0.9; *P* = .005; Table 3C). Increasing APR-DRG severity also significantly decreased odds of discharge to home (aOR = 0.62, 95% CI 0.6, 0.7; *P* < .001). Hospitalist model type also retained its significance with in-hospital mortality in a linear regression (aOR = 1.89, 95% CI 1.14, 3.23; *P* = .02). We note that, in this model, APR-DRG severity conferred twofold higher odds of in-hospital mortality (aOR = 2.05, 95% CI 1.78, 2.36; *P* < .001). See Table 3D for additional details.

No significant differences were found in relevant satisfaction scores between the two models as reported by Press Ganey. About 81% of patients treated under the PA-physician model reported feeling that physicians treated them with courtesy and respect, compared with 79.2% of physician-only model patients (*P* = .53). Similar results were found for whether physicians explained things in a way that patients could understand (69.9% PA-physician model, 64.8% physician-only model; *P* = .3) and whether patients felt that physicians listened carefully (69.1% PA-physician model, 68% physician-only model; *P* = 1).

## DISCUSSION

The PA-physician hospitalist model at a community hospital showed equal to superior results compared with the traditional physician-only hospitalist model. Compared with the traditional physician-only model, the PA-physician model demonstrated more discharges home than to skilled nursing facilities and a shorter LOS without compromising readmission rates. Although these metrics are inherently imperfect in capturing the success of any hospitalist program, these findings support the efficacy of the PA-physician hospitalist model established at our institution. In our dataset, LOS was tabulated in hours or days. However, the data in days format were drawn from a crude difference between admit date and discharge date in whole numbers, lacking actual times of admission or discharge. For greater precision, we opted to capture LOS in hours. No difference was found in the qualitative result between the two tabulations and thus the difference in LOS hours represents the average net result of patients both leaving earlier in the same day and those leaving one or more actual days sooner. In usual quality improvement initiatives, this type of average decrease in total hours is felt to be meaningful as any fractional improvement in this key metric has major cost and quality implications. Although greater efficiency in the PA-physician model in discharging patients earlier in the same day could account for a portion of the overall difference in LOS, this nevertheless would be meaningful because it would represent increased bed turnover. This potentially reduces the time admissions are boarded in the ED, and can affect staffing assignments and other resource use. Because LOS represents the combined result of multiple factors, employing a PA-physician model may be one important contributor to systems seeking to improve LOS.

More patients were discharged home rather than to skilled nursing or other facilities with the PA-physician model, with no significant differences in readmission rates. This shows an additional tangible monetary benefit, as sending a Medicare patient home with home healthcare rather than to a skilled nursing facility may save insurers (including Medicare) more than $4,000 per patient.^11^ Having a higher number of patients discharged to home rather than to another facility also may reduce downstream readmission rates and improve patient satisfaction.

A possible explanation for lower LOS and more discharges home in our PA-physician model is that PAs were able to spend more time on care management and coordinating discharges with patients than their physician counterparts, because each PA saw fewer patients per day than were seen in the traditional physician-only model. Our PA-physician hospitalist model relies on a team approach. Each PA sees a comparatively smaller number of patients, allowing more opportunity to focus on the details of the medical case, as well as the social factors to coordinate case management and improve hospital throughput. The physician in this model can focus more on medicine and the most complex cases. Patients benefit from two clinicians caring for them, and therefore more clinician visits and attention. Throughout the study period, physicians were required to see every patient every day with few exceptions—for example, a patient could be discharged by the PA if the patient had been seen and admitted by a physician within 24 hours before discharge and if the discharge was approved by the physician. After the study period, however, hospital bylaws independently changed to allow PA-only visits, and the physician was no longer required to physically see each patient every day. This has further allowed the physician to focus efforts on collaborating with PAs on more complex medical cases that could use the skills of two competent clinicians, and simply reviewing plans with the PA for more stable patients.

APR-DRG severity weights were significantly higher in the 2017 PA-physician model; however, the difference was quite small and multiple factors may have played a role, including an early 2017 training session that taught clinicians how to bill more appropriately. In-hospital mortality was significantly lower in the PA-physician model; however, the number of deaths was quite low in both groups (less than 3% of cases), which may affect the assessment of this difference. The circumstances in which in-hospital deaths occurred were not examined in our anonymized dataset, leaving us unable to evaluate the clinical significance in the discrepancies between these deaths under these two models.

Our model also used PAs who completed a fellowship in hospitalist medicine and our model clearly relies on a solid PA-physician team and mutual trust. As only one PA in 2017 did not come through the hospitalist fellowship, this study did not compare fellowship trained with nonfellowship trained PAs. This specific training and experience likely played a role in the results we see, and the quality of PA training should be taken into account when creating such practice models.

## LIMITATIONS

This is a small retrospective, nonrandomized study, evaluated at one urban community hospital. Two physicians and six PAs made up the majority of the PA-physician model analyzed in this study, which potentially limits the generalizability of the results. Encouragingly, comparable results have been found with similar models at academic medical centers when compared with resident models.^3^,^7^

These data also are incomplete with respect to consultations and observation admissions, which are included in routine hospitalist duties under both models but could not be assessed in our analysis. Comparing different time periods has its own challenges: populations change, system-wide efforts focus more on decreasing LOS and readmissions over time, and clinicians become more proficient at billing and coding with directed training.

There may be some negligible physician-model admixture in the 2017 PA-physician model dataset because physician-only hospitalists cared for patients during the PA-physician teams' scheduled breaks. That is, some patients may have been cared for under the physician-only model and handed off to the PA-physician team, and vice versa.

Press Ganey scores are only captured according to billing clinician and the physicians performed all billing during the study period. This prevented us from achieving granularity on individual PA performance. However, as the physicians over the study saw every PA patient, these scores should represent the same patient subset and the team as a whole.

## CONCLUSIONS

Our findings comparing a PA-physician hospitalist model with a traditional physician-only hospitalist model support the efficacy of our PA-physician hospitalist program. The findings are consistent with those of other studies assimilating different PA hospitalist models.^3^-^5^,^7^ We developed our staffing model of three PAs to one physician as a salary-neutral model compared with physician-only hospitalist practice. Being salary-neutral on face, the cost savings of our PA-physician model, in light of decreased LOS and increased discharges to home, would be expected to result in a significant net savings to the hospital system and healthcare system. This team-based approach lets PAs take primary responsibility for patients of all acuity levels, while the overseeing physician has more time to spend on the most complex cases. Future studies are warranted to further evaluate staffing models for PA hospitalists in multiple types of hospitals in order to determine the ideal model.
